# Tillage and seeding strategies for wheat optimizing production in harvested rice fields with high soil moisture

**DOI:** 10.1038/s41598-020-80256-7

**Published:** 2021-01-08

**Authors:** Jinfeng Ding, Fujian Li, Tao Le, Dongyi Xu, Min Zhu, Chunyan Li, Xinkai Zhu, Wenshan Guo

**Affiliations:** 1 Jiangsu Key Laboratory of Crop Genetics and Physiology, and Jiangsu Key Laboratory of Crop Cultivation and Physiology, Agricultural College of Yangzhou University, Yangzhou, 225009 Jiangsu China; 2 Co-Innovation Center for Modern Production Technology of Grain Crops, Yangzhou University, Yangzhou, 225009 Jiangsu China

**Keywords:** Plant sciences, Environmental impact

## Abstract

Suitable tillage and seeding strategies for wheat can be used to combat excessive residues and poor soil conditions in harvested rice fields. This study investigated the effects of different tillage (zero tillage and rotary tillage) and seeding methods on wheat growth, grain yield, nitrogen (N) uptake and utilization, and economic benefit when the soil moisture was high during the tillage and seeding practices. In 2016–2017, three seeders were tested: SM1-1, SM2, and SM3; in 2017–2018, four seeders were tested: SM1-2, SM2, SM3, and SM4. Although the soil moisture was different between years, zero tillage could be used to reduce the sowing depth, which facilitated early-phase wheat growth and N uptake compared with rotary tillage, resulting in higher grain yield, NUpE, and net return. In 2016–2017 (high wet soil), a small-size seeder (SM1-1) with sowing near the soil surface facilitated higher grain yield, NUpE, and net returns compared with the other seeders; in 2017–2018 (low wet soil), medium-size seeders (SM3 and SM4) were more suitable than small-size seeders (SM1-2 and SM2). In both years, the seeders that performed the best mainly improved the spike numbers while increasing N uptake, especially after anthesis. Zero tillage lowered input costs, but small-size seeders did not reduce costs due to the higher labor costs associated with their low working efficiency. Improving net returns depends largely on increasing yield. In conclusion, zero tillage is recommended for wheat production in harvested rice fields with a high soil moisture content, but the suitable seeding method needs to be confirmed according to the soil moisture content.

## Introduction

Adopting suitable soil tillage and sowing methods, which are the initial steps of wheat planting, can facilitate growth and yield. Choosing the most suitable methods depends on multiple factors, especially soil water^1–4^. Baiamonte et al.^1^ reported that no-tillage is a suitable management option under rainfed conditions, but there is the probability that the yield is affected by the cropping system, residue management, and the aridity index. Results presented by Ali et al. showed that different cultivation modes under different precipitation levels significantly boosted wheat growth and yield in a dry-land farming system^2^. However, tillage and sowing strategies for wheat production in high-rainfall areas, and specifically for adopting a rice–wheat rotation system (RWRS), have been rarely reported.

Rice–wheat planting systems are mainly located in the middle and lower reaches of the Yangtze River Basin (YRB) in China and in the Indo-Gangetic Plains (IGP). These systems provide large amounts of cereals for the local area. Due to the conventional use of flooded conditions for growing rice in these regions, the soil is easily water-saturated^5^. Furthermore, the soil has become more sticky and heavier due to long-term soil puddling before rice is transplanted^6–8^. With improvements in rice yield, an increased amount of rice straw is incorporated into the fields, which has a seriously adverse effect on wheat seedling emergence and growth^9^. Soil tillage has been adopted to address these problems. Soil tillage can break the hardpan and mix residues and soil to improve soil porosity and promote root development and plant growth^10–13^. However, under a rice–wheat rotation, the flooding practices for rice planting typically end approximately 10 days before rice is harvested in the YRB area. In order to save water and ensure sufficient water for rice growth, there is almost no drainage system in the rice fields. Therefore, after the rice is harvested, the soil needs an extended period for the moisture content to decrease; rainfall at this time leads to poor soil conditions. Under high soil moisture, tillage can result in soft and sticky soil, which can cause seeders to sink into the soil, seeding ports to block, and seeds to be trapped by the soil block. However, waiting for moisture reduction wastes solar thermal resources.

In the IGP, zero tillage with rice-straw mulching have been adopted for early sowing of wheat and high profitability by reducing tillage costs. Wheat yields produced under this method are not lower than the yields obtained under the conventional method of tillage with later sowing^6,14,15^. Although zero tillage maintains soil structural stability and reduces water loss, which can promote plant growth, especially under drought conditions^4,16^, further study is required to elucidate whether zero tillage is an effective strategy under high soil moisture. Paul et al.^17^ indicated that zero tillage inhibited the growth of sunflower plants due to decreases in soil water content and solute potential in the surface soil layers in a wet clay soil.

In the YRB area, mechanical seeders have primarily been used for wheat sowing, and various types of machine have been developed, including a small-size seeder (~ 12 HP), medium-size seeder (~ 85 HP), and large-size seeder (with higher HP). The small- and medium-size seeders have been used to sow most of the arable land in this area. If classified by seed arrangement, the seeders can be divided into broadcast, drill (narrow seed line width), and strip (wide seed line width) seeders. Furthermore, with the development of seeding machines, an increasing number of seeders that are modified for better-adapting soil and production conditions are becoming available on the market and are being tested. The effects of sowing depth, row spacing, and row width on wheat growth, yield, and quality have been reported^18–21^. However, optimal sowing technologies for promoting wheat production and mechanical sowing strategies for facilitating the achievement of suitable sowing conditions have rarely been reported. Sidhu et al*.*^22^ reported the adoption of the Turbo Happy Seeder for sowing wheat into heavy rice residues in north-western India, but it was unable to operate in wet straw.

In practical production, the choice of suitable mechanical tillage and sowing methods conventionally depends on the working efficiency, fuel cost, and grain yield, which largely determines the economic benefit. However, due to the lack of experimental comparisons, there are various experimental tillage and sowing combinations that are used to address specific soil conditions and the return of straw residues while achieving high yields and benefits. These include zero tillage or tillage, followed by sowing with different types of small- or medium-size seeders. The present study was designed to: (1) investigate the effects of tillage methods and mechanical sowing methods on wheat growth, grain yield, N uptake and utilization, as well as the economic benefit, and (2) propose an optimal tillage and sowing combination to achieve high yield and profitability in the YRB area. Due to high rainfall before wheat sowing in both experimental years, there was the potential to explore tillage and seeding strategies for wheat production in harvested rice fields with high soil moisture. Thus, the results of this study can expand upon the knowledge of wheat cultivation in RWRSs.

## Materials and methods

### Experimental location and ecological conditions

**Figure 1:**
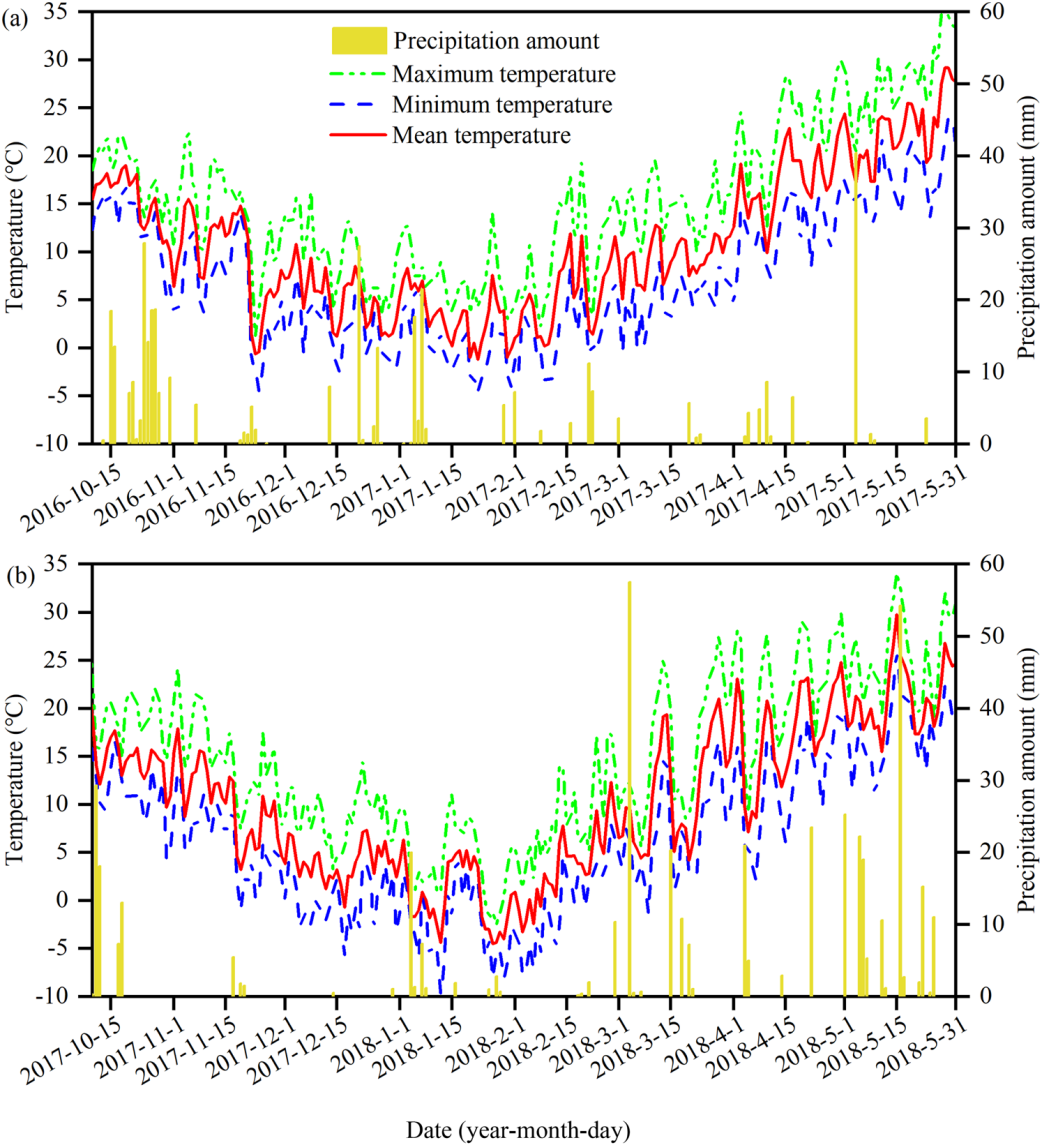
Daily mean, minimum, and maximum temperature and precipitation amount during the wheat-growing seasons of 2016–2017 and 2017–2018 (**a** and b).

Field experiments were carried out in Sihong city (Jiangsu, China) during the wheat seasons of 2016–2017 (2017) and 2017–2018 (2018). The study site is located in the typical RWRS region of the YRB with a humid north subtropical monsoon climate. The daily mean, minimum, and maximum temperature and the precipitation during the two wheat-growing seasons, which were provided by the local meteorological station, are shown in Fig. 1.

The return of rice and wheat residues to the experimental field has been conducted for 10 years. Conventional planting technologies for rice include puddling, transplanting, and flooding. The soil in the study area is a clay according to the USDA (the U.S. Department of Agriculture) classification and contains 22% sand, 18% silt, and 60% clay. The soil was sampled at 0–20 cm after the rice harvest to measure the nutrient status. The soil contained 30.6 g kg^−1^ organic C, 121.3 mg kg^−1^ available N, 25.9 mg kg^−1^ available P, and 180.0 mg kg^−1^ available K in 2016 and 27.9 g kg^−1^ organic C, 116.7 mg kg^−1^ available N, 33.9 mg kg^−1^ available P, and 78.4 mg kg^−1^ available K in 2017. The gravimetric soil water content, which was measured during tillage and seeding, was at 95% field capacity in 2016 and at 84% in 2017. The high soil moisture was attributed to very high rainfall in October, when rice was at the grain-filling and maturity stages, and a late ending of the flooding practice for rice planting (Fig. 1).

### Experimental design and management

The experimental process included rice threshing, shredding, and the spraying of residues (using a combine harvester), fertilizer application, tillage, and seeding. The rice residue incorporated into the field was approximately 8.2 t ha^−1^ in both years, the length of shredded straws was approximately 8 cm, and the height of the stubble was approximately 8 cm.

The prepared field was separated into three blocks (replicates). Each block was then split into two parts (main plots). One plot was subjected to rotary tillage twice (with an approximate tillage depth of 15 cm), and the other was maintained without tillage (zero tillage). Three (2017) or four (2018) types of seeders were used for seeding (subplots). The seeders included a small-size broadcast seeder (SM1-1), a small-size strip seeder (SM2), and a medium-size strip seeder (SM3) in 2017. In 2018, a small-size drill seeder (SM1-2), SM2 and SM3 seeders, and a medium-size drill seeder (SM4) were used. SM1-1 and SM1-2 involved the same seeder, but the seed pipe of the SM1-1 seeder was removed due to moist soil blocking the seeding port in 2017. As a result, seeds were evenly distributed in the field after seeding, similar to broadcast sowing. The type, operation procedure, and features of these seeders are shown in Table 1. The two tillage methods were represented by the two main plots, and the various seeding methods were subplots using a split-plot design. The average area of the subplots was 118 m^2^, with slight differences according to the working range of the seeders.

**Table 1:** Type, operation procedure, and features of the seeders.

Seeding method	Seeder type (model)	Operation procedure	Working range (mm)	Row spacing (mm)	Drilling width (mm)	Sowing depth	Power matched (HP)
SM1-1	Small-size broadcast seeder (2BG-6A)	Shallow no-inverse rotary, sowing, roll compaction	1200			Near soil surface	12
SM1-2	Small-size drill seeder (2BG-6A)	Shallow no-inverse rotary, sowing, roll compaction	1200	200	10	Near soil surface	12
SM2	Small-size strip seeder (2BG-6A)	Sowing, shallow no-inverse rotary, roll compaction	1200	200	80	~ 1 cm	12
SM3	Medium-size strip seeder (2BMQF-7/14)	Shallow inverse rotary, sowing, shallow no-inverse rotary, press wheel compaction	2180	250	100	~ 2 cm	85
SM4	Medium-size drill seeder (2BFGK-10(8)230)	Shallow inverse rotary, sowing, shallow no-inverse rotary, roll compaction	2300	180	10	~ 2 cm	85

*SM1-1* small-size broadcast seeder, *SM1-2* small-size drill seeder, *SM2* small-size strip seeder, *SM3* medium-size strip seeder, *SM4* medium-size drill seeder.

Yangmai23 is a winter wheat cultivar that is widely planted in the YRB area. Yangmai23 seeds were sown on November 12, 2016, and November 6, 2017, at approximately 160 kg ha^−1^. At the three-leaf stage, three areas of 1 m × 1 m were selected in each subplot, and 270 seedlings per square meter were retained via manual removal. Nitrogen fertilizer was applied at the stages of pre-sowing, five-leaf, three-leaf remaining, and flag leaf visible, at rates of 120, 24, 48, and 48 kg ha^−1^, respectively. In addition, 72 kg ha^−1^ of phosphorus (P_2_O_5_) and potassium (K_2_O) fertilizers were separately applied at the pre-sowing and three-leaf remaining stages. Due to abundant rainfall, we did not need to irrigate at all during the two growing seasons. The harvest date was May 25 in both 2017 and 2018. Details of the management in this area have been specified in the report by Ding et al*.*^23^

### Sampling and measurements

#### Maximum tiller number, spike number, tiller fertility, and grain yield

In the areas in which the seedling number was fixed, the tillers were counted at the stem elongation stage to obtain the maximum tiller number, and the number of spikes was counted in the field at the maturity stage. The spikes in these areas were manually harvested after the count of spikes using garden shears, followed by threshing and weighing to calculate the grain yield. The moisture content of the threshed grains was measured by a Grain Analyzer (Infratec 1241, Foss, Denmark). Tiller fertility refers to the ratio of the number of efficient tillers to the maximum tiller number. The single spike yield was calculated as the grain yield divided by the spike number. Grain yield and single spike yield were adjusted to 13% moisture content.

#### Nitrogen accumulation and nitrogen uptake efficiency (NUpE)

At the anthesis and maturity stages, 40 culms were continuously sampled from the plots, which had a fixed planting density. The culms were dried in ovens at 70 °C after being separated into spikes and other organs. The mature spikes were threshed by hand into grains and other parts. The dry mass of these samples was measured after oven-drying, and the N concentration was determined using the indophenol blue method. N accumulation was calculated based on the dry mass and N concentration. The pre-anthesis and total N accumulation represented the accumulation at anthesis and maturity, respectively. The post-anthesis N accumulation represented the difference in the N accumulation between that at anthesis and maturity. The NUpE (kg kg^−1^) was defined as the increased total N accumulation caused by N application, divided by the applied N rates.

#### Working efficiency and economic analysis

Over the course of the experiments, the amounts of various inputs (seeds, fertilizers, labor, mechanical operations, and agricultural chemicals) were recorded, and their local prices were investigated to provide information on the cost. The total cost did not include the field rental expenses. The total cost was calculated as follows:

$${\text{Total}}\,{\text{cost }} = {\text{ seeds}}\,{\text{costs }} + {\text{ fertilizers}}\,{\text{costs }} + {\text{ biotic}}\,{\text{stress}}\,{\text{control}}\,{\text{costs }} + {\text{ labor}}\,{\text{costs }} + {\text{ tillage}}\,{\text{costs }} + {\text{ seeding}}\,{\text{costs }} + {\text{ harvest}}\,{\text{costs}},$$

where seeds costs, fertilizers costs, and labor costs (without considering the labor costs of the tractor driver) were calculated as inputs multiplied by the price. A professional agro-management organization provided tillage, chemical application, and grain harvesting services at a regulated price. Therefore, the various seeding methods and annual differences in the tillage price accounted for the differences in the total cost between the two years, although there were slight changes in other costs that were not considered.

The costs of seeding were calculated individually, depending on the records of the labor price, working time, and fuel costs:

$${\text{Seeding}}\,{\text{costs }} = {\text{ working}}\,{\text{time }} \times {\text{ labor}}\,{\text{cost}}\,{\text{of}}\,{\text{the}}\,{\text{tractor}}\,{\text{driver }} + {\text{ fuel}}\,{\text{consumption }} \times {\text{ fuel}}\,{\text{price}},$$

where the labor cost of the tractor driver of small-size seeders was lower than that of medium-size seeders. The working time and fuel consumption were recorded at seeding.

A minimum grain purchase price formulated by the state was 2.36 yuan (CNY) kg^−1^ in the study year. The gross returns were obtained as follows:

Gross returns = grain yield × grain purchase price.

Net returns were calculated as follows:

Net returns = gross returns—total costs.

The benefit–cost ratio was calculated as follows:

Benefit–cost ratio = net returns/total costs.

In the defined areas, the working time and fuel consumption of each seeder were individually recorded. According to the working time and seeding area, we calculated the working efficiency:

Working efficiency = seeding area/working time.

Due to being restricted by the seeding area, there was only one investigation into the costs of the different seeding methods, and the differences caused by the tillage method were not considered. However, the sowing area under the various tillage methods was the same for each seeder.

### Statistical analysis

The software used for analyzing the data was the DPS v7.05 system (Shanghai, China). Analysis of variance (ANOVA) was used to determine the significance of differences between treatments, while Fisher’s least significant difference (LSD) posthoc test (*p* < 0.05) was used to analyze the differences.

## Results

### Grain yield and yield components

In both experimental years, tillage and seeding significantly affected the grain yield. However, there was only a significant interaction between these factors in 2018 (Table 2). In both years, the grain yield was significantly higher under zero tillage than under rotary tillage. These differences in grain yield were due to the wide differences in single spike yield in 2017, but in spike number in 2018 (Table 3). The different seeding methods resulted in changes in grain yield that were mainly attributed to the effects of spike number. An exception to this was in 2018, when SM1-2 resulted in a significantly higher single spike yield than SM2. Under the growing conditions of 2017, SM1-1 achieved the highest grain yield and spike number among all the seeding methods; the spike number and grain yield were similar between SM2 and SM3. In 2018, the seeding methods that resulted in the highest spike number and grain yield were SM3 and SM4, followed by SM1-2. The differences in grain yield and spike number between SM3, SM4, and SM1-2 were not marked under zero tillage. However, under rotary tillage, SM3 and SM4 led to significantly higher grain yield and spike number than SM1-2 (Table 4).

**Table 2:** Analysis of variance (ANOVA) *p*-values for the effects of tillage method, seeding method, and their interaction (T × S), on the maximum tiller number, tiller fertility, grain yield, and yield components in 2017 and 2018.

Year	Treatment	Maximum tiller number	Tiller fertility	Spike number	Single spike yield	Grain yield
2017	Tillage method (T)	0.054	0.023	0.901	0.040	0.020
	Seeding method (S)	< 0.001	0.041	< 0.001	0.069	< 0.001
	T × S	0.765	0.307	0.221	0.419	0.194
2018	Tillage method (T)	0.008	0.366	0.019	0.707	0.015
	Seeding method (S)	< 0.001	< 0.001	< 0.001	0.083	< 0.001
	T × S	< 0.001	< 0.001	0.023	0.362	0.001

**Table 3:** The effects of the tillage method and seeding method on the maximum tiller number, tiller fertility, grain yield, and yield components in 2017 and 2018.

Year	Treatment	Maximum tiller number (m^−2^)	Tiller fertility (%)	Spike number (m^−2^)	Single spike yield (g)	Grain yield (t ha^−1^)
2017	Tillage method					
	Rotary tillage	1006 ± 39 a	34 ± 1 b	521 ± 22 a	1.27 ± 0.02 b	6.59 ± 0.20 b
	Zero tillage	907 ± 40 a	39 ± 1 a	520 ± 25 a	1.39 ± 0.03 a	7.22 ± 0.28 a
	Seeding method					
	SM1-1	1047 ± 50 a	39 ± 3 a	573 ± 3 a	1.30 ± 0.07 a	7.43 ± 0.42 a
	SM2	924 ± 44 b	34 ± 3 b	494 ± 7 b	1.31 ± 0.04 a	6.48 ± 0.28 b
	SM3	899 ± 56 b	36 ± 1 ab	495 ± 12 b	1.38 ± 0.08 a	6.81 ± 0.24 b
2018	Tillage method					
	Rotary tillage	891 ± 48 b	29 ± 5 a	447 ± 27 b	1.36 ± 0.04 a	6.07 ± 0.39 b
	Zero tillage	1197 ± 44 a	27 ± 3 a	518 ± 17 a	1.34 ± 0.02 a	6.93 ± 0.20 a
	Seeding method					
	SM1-2	1154 ± 111 a	19 ± 5 c	443 ± 67 b	1.43 ± 0.04 a	6.32 ± 0.79 b
	SM2	1016 ± 226 bc	24 ± 4 b	441 ± 27 b	1.29 ± 0.04 b	5.69 ± 0.58 c
	SM3	960 ± 90 c	36 ± 1 a	518 ± 27 a	1.36 ± 0.03 ab	7.03 ± 0.20 a
	SM4	1047 ± 185 b	34 ± 5 a	527 ± 22 a	1.32 ± 0.02 ab	6.97 ± 0.16 a

Data are presented as means ± standard errors. Different letters indicate significant differences between treatments at the 0.05 probability level. *SM1-1* small-size broadcast seeder, *SM1-2* small-size drill seeder, *SM2* small-size strip seeder, *SM3* medium-size strip seeder, *SM4* medium-size drill seeder.

**Table 4:** Effects of tillage and seeding method combinations on wheat (a) maximum tiller number, (b) tiller fertility, (c) spike number, and (d) grain yield in 2018.

Tillage method	Seeding method	Maximum tiller number (m^−2^)	Tiller fertility (%)	Spike number (m^−2^)	Grain yield (t ha^−1^)
Rotary tillage	SM1-2	1043 ± 26 b	14 ± 1 e	377 ± 10 d	5.53 ± 0.12 d
	SM2	790 ± 15 c	28 ± 2 c	413 ± 8 d	5.11 ± 0.18 e
	SM3	870 ± 24 c	37 ± 2 a	491 ± 17 c	6.83 ± 0.28 b
	SM4	862 ± 46 c	40 ± 1 a	505 ± 19 bc	6.81 ± 0.29 b
Zero tillage	SM1-2	1264 ± 70 a	24 ± 2 cd	510 ± 20 abc	7.11 ± 0.14 ab
	SM2	1243 ± 5 a	20 ± 1 d	468 ± 14 c	6.27 ± 0.20 c
	SM3	1050 ± 20 b	35 ± 2 ab	545 ± 21 ab	7.23 ± 0.10 a
	SM4	1232 ± 65 a	29 ± 2 bc	548 ± 6 a	7.12 ± 0.10 ab

Data are presented as means ± standard errors. Different letters indicate significant differences between treatments at the 0.05 probability level. *SM1-2* small-size drill seeder, *SM2* small-size strip seeder, *SM3* medium-size strip seeder, *SM4* medium-size drill seeder.

### Tiller number and tiller fertility

In 2017, zero tillage resulted in a higher tiller fertility than rotary tillage, despite the similar maximum tiller number. However, in 2018, the maximum tiller number was significantly greater under zero tillage, but zero tillage and rotary tillage resulted in a similar tiller fertility (Table 3). In both years, the seeding method significantly affected the maximum tiller number and tiller fertility, and in 2018 these differences were dependent on the tillage method (Table 2). SM1-1 and SM1-2 achieved a significantly higher maximum tiller number than the other seeding methods in the two years; SM3 resulted in a relatively lower maximum tiller number. Under zero tillage in 2018, SM1-2, SM2, and SM4 resulted in similar maximum tillage numbers, but under rotary tillage, SM1-2 had the most tillers, with insignificant differences between the other seeding methods (Table 4). The results also showed that SM1-1 was better at facilitating tiller fertility than the other methods in 2017 (Table 3). However, in 2018, the tiller fertility was better in the SM3 and SM4 treatments, despite being relatively lower using SM4 under zero tillage (Table 4).

### Nitrogen accumulation and NUpE

Compared with rotary tillage, zero tillage increased pre-anthesis and total N accumulation, as well as NUpE, in both years. However, tillage did not affect post-anthesis N accumulation (Tables 5 and 6). The different seeding methods resulted in significant changes in N accumulation and NUpE. Compared with SM2 and SM3 in 2017, SM1-1 resulted in higher pre-anthesis, post-anthesis, and total N accumulation, as well as higher NUpE (with insignificant differences between SM2 and SM3). In 2018, pre-anthesis N accumulation was significantly higher under SM1-2 and SM4 than under SM2. Although pre-anthesis N accumulation under SM1-2 was not greatly different from under SM3 and SM4, the latter two methods promoted more post-anthesis N uptake than the other methods while achieving higher total N accumulation and NUpE.

**Table 5:** Analysis of variance (ANOVA) *p*-values for the effects of tillage method, seeding method, and their interaction (T × S), on nitrogen accumulation, nitrogen uptake efficiency (NUpE), and the net returns in 2017 and 2018.

Year	Treatment	Nitrogen accumulation			NUpE	Net returns
		Pre-anthesis	Post-anthesis	Total		
2017	Tillage method (T)	0.011	0.353	0.019	0.005	0.014
	Seeding method (S)	0.006	0.006	< 0.001	0.001	< 0.001
	T × S	0.346	0.830	0.296	0.237	0.194
2018	Tillage method (T)	0.042	0.947	0.034	0.042	0.011
	Seeding method (S)	0.048	0.006	< 0.001	< 0.001	< 0.001
	T × S	0.017	0.267	0.014	0.002	0.001

**Table 6:** The effects of the tillage method and seeding method on nitrogen accumulation, nitrogen uptake efficiency (NUpE), and the net returns in 2017 and 2018.

Year	Treatment	Nitrogen accumulation (kg ha^−1^)			NUpE (kg kg^−1^)	Net returns (yuan ha^−1^)
		Pre-anthesis	Post-anthesis	Total		
2017	Tillage method					
	Rotary tillage	139 ± 5 b	44 ± 2 a	183 ± 8 b	37.9 ± 1.7 b	7566 ± 446 b
	Zero tillage	157 ± 3 a	47 ± 4 a	204 ± 6 a	40.9 ± 1.3 a	9353 ± 637 a
	Seeding method					
	SM1-1	157 ± 6 a	52 ± 2 a	209 ± 8 a	42.7 ± 1.1 a	9635 ± 1149 a
	SM2	143 ± 11 b	39 ± 1 b	182 ± 12 b	37.7 ± 0.8 b	7522 ± 819 c
	SM3	144 ± 10 b	45 ± 2 b	189 ± 11 b	37.8 ± 2.6 b	8223 ± 713 b
2018	Tillage method					
	Rotary tillage	154 ± 4 b	47 ± 2 a	201 ± 5 b	31.9 ± 2.7 b	6327 ± 922 b
	Zero tillage	165 ± 1 a	46 ± 3 a	211 ± 3 a	37.9 ± 1.5 a	8666 + 460 a
	Seeding method					
	SM1-2	161 ± 8 a	40 ± 1 b	201 ± 9 b	32.7 ± 4.7 b	7039 ± 2016 b
	SM2	153 ± 10 b	43 ± 4 b	196 ± 7 b	30.2 ± 3.5 b	5627 ± 1511 c
	SM3	160 ± 4 ab	52 ± 3 a	212 ± 7 a	37.5 ± 4.5 a	8747 ± 631 a
	SM4	163 ± 1 a	51 ± 1 a	214 ± 1 a	39.5 ± 0.7 a	8572 ± 519 a

Data are presented as means ± standard error. Different letters indicate significant differences between treatments at the 0.05 probability level. *SM1-1* small-size broadcast seeder, *SM1-2* small-size drill seeder, *SM2* small-size strip seeder, *SM3* medium-size strip seeder, *SM4* medium-size drill seeder.

Interactions between tillage method and seeding method were found to have a significant effect only on the total and pre-anthesis N accumulation and NUpE in 2018 (Table 5). Under zero tillage, pre-anthesis and total N accumulation were not greatly different under SM1-2, SM3, and SM4. However, under rotary tillage, the pre-anthesis and total N accumulation were significantly higher under SM3 and SM4 than under SM1-2 and SM2 (except for an insignificant difference in pre-anthesis N accumulation between SM1-2 and SM3; Table 7). With respect to NUpE, SM4 resulted in the greatest NUpE under rotary tillage, followed by SM3. However, under zero tillage, SM3 and SM4 resulted in similar NUpE values, which were higher than those produced by the other seeding methods (Table 7).

**Table 7:** The effects of tillage and seeding method combinations on (a) pre-anthesis and (b) total nitrogen accumulation and (c) nitrogen uptake efficiency (NUpE) in 2018.

Tillage method	Seeding method	Pre-anthesis nitrogen accumulation (kg ha^−1^)	Total nitrogen accumulation (kg ha^−1^)	NUpE (kg kg^−1^)
Rotary tillage	SM1-2	153 ± 4 cd	192 ± 3 d	28.0 ± 1.4 e
	SM2	143 ± 4 d	190 ± 6 d	26.7 ± 0.4 e
	SM3	156 ± 5 bc	205 ± 3 bc	32.9 ± 0.2 d
	SM4	164 ± 2 ab	215 ± 6 ab	40.1 ± 0.2 ab
Zero tillage	SM1-2	169 ± 6 a	210 ± 2 abc	37.4 ± 0.1 bc
	SM2	164 ± 5 ab	203 ± 4 c	33.7 ± 1.3 cd
	SM3	164 ± 4 ab	219 ± 1 a	42.0 ± 1.8 a
	SM4	162 ± 5 abc	213 ± 4 ab	38.8 ± 1.6 ab

Data are presented as means ± standard errors. Different letters indicate significant differences between treatments at the 0.05 probability level. SM1-2 small-size drill seeder, SM2 small-size strip seeder, SM3 medium-size strip seeder, SM4 medium-size drill seeder.

### Economic input and output

**Figure 2:**
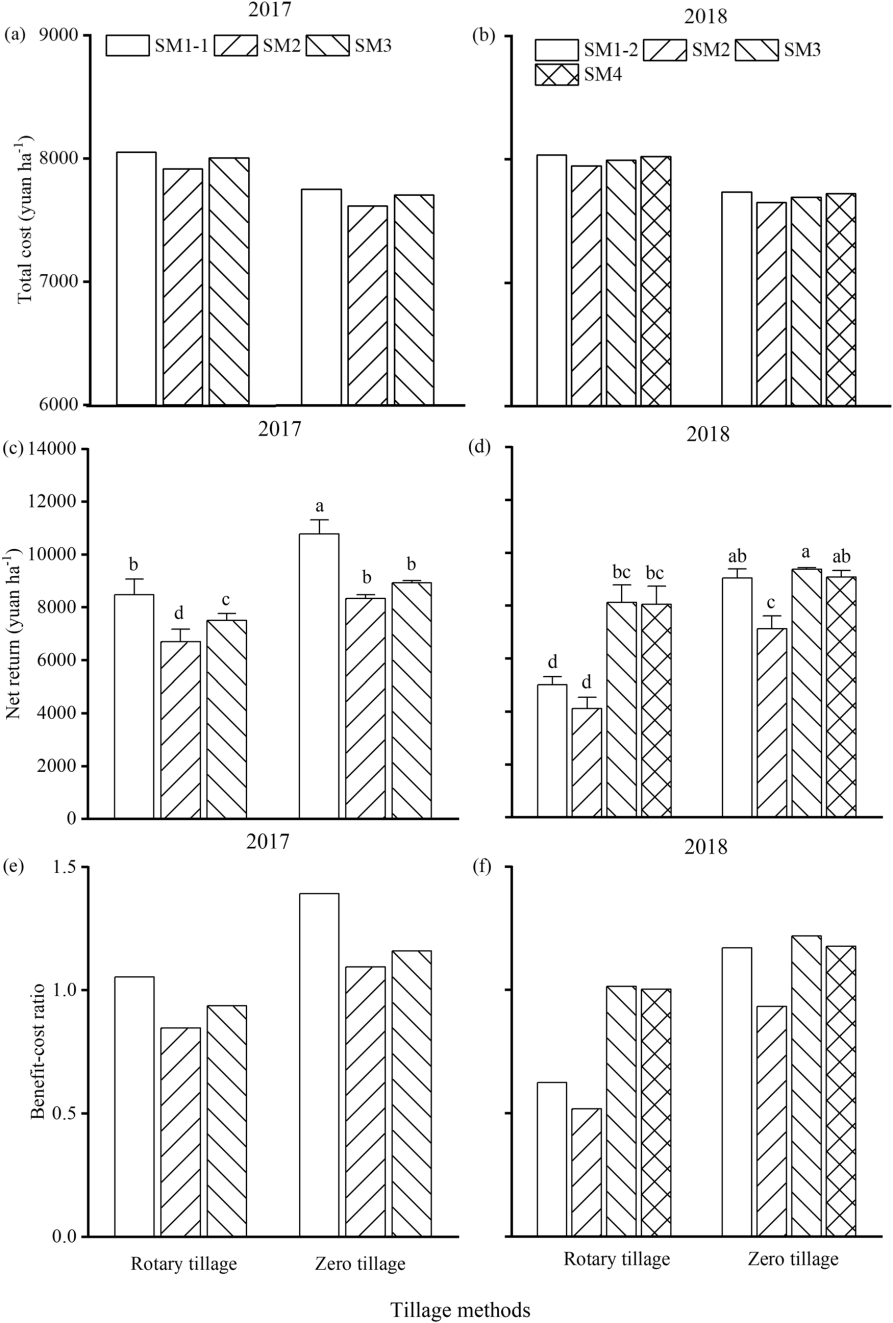
The total costs (**a** and **b**), net returns (**c** and **d**), and benefit–cost ratio (**e** and **f**) of production under various tillage and seeding methods in 2017 (**a**,**c**, and **e**) and 2018 (**b**,**d**, and **f**). The bars indicate the standard error. Different letters above the bars indicate significant differences between treatments at the 0.05 probability level. *SM1-1* small-size broadcast seeder, *SM1-2* small-size drill seeder, *SM2* small-size strip seeder, *SM3* medium-size strip seeder, *SM4* medium-size drill seeder.

In the two wheat-growing seasons, zero tillage significantly improved net returns (by 23.7% in 2017 and 49.2% in 2018) compared to rotary tillage (Tables 5 and 6). This higher net return was attributed to the saved tillage inputs (Fig. 2a,b) and the increased grain yields under zero tillage (Table 3). The seeding method also greatly affected the net returns. Among all the methods, SM1-1 resulted in the greatest net return followed by SM3 in 2017, and SM3 and SM4 resulted in the highest return, followed by SM1-2 in 2018. However, the effects of the seeding methods on the net returns depended on the tillage methods in 2018. Under rotary tillage, SM3 and SM4 facilitated greater net returns than the other seeding methods. However, SM3, SM4, and SM1-2 achieved similar net returns under zero tillage and significantly higher net returns than SM2 (Fig. 2d).

**Figure 3:**
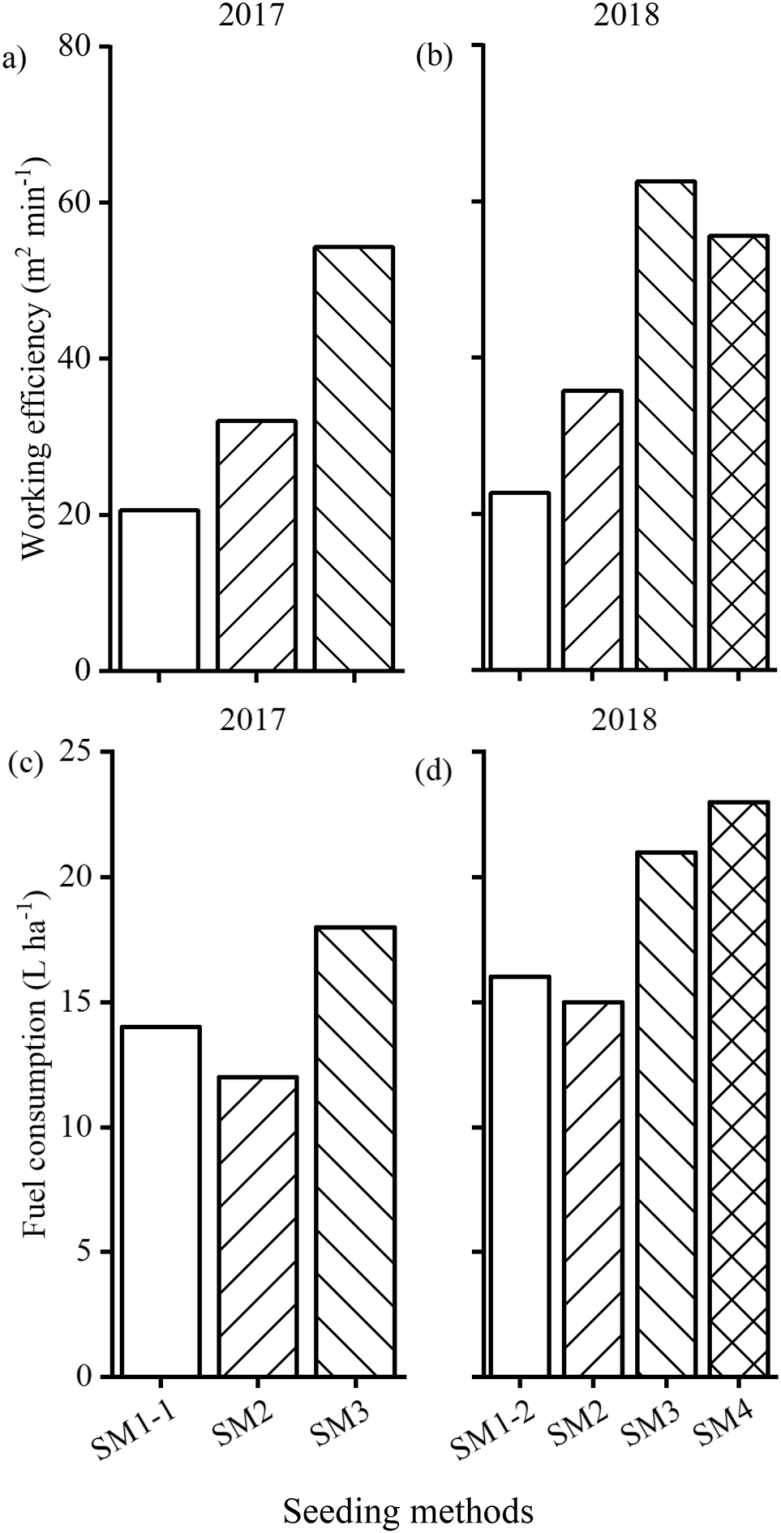
The working efficiency (**a** and **b**) and fuel consumption (**c** and **d**) of the different seeders in 2017 (**a** and **c**) and 2018 (**b** and **d**). *SM1-1* small-size broadcast seeder, *SM1-2* small-size drill seeder, *SM2* small-size strip seeder, *SM3* medium-size strip seeder, *SM4* medium-size drill seeder.

Through cost analysis, it was found that the various seeding methods resulted in very slight differences in total costs (Fig. 2a,b). As shown in Fig. 3, the seeding methods with low working efficiency, such as SM1-1 (SM1-2) and SM2 (small-size seeders), were accompanied by low fuel costs. However, the methods with high fuel consumption, such as SM3 and SM4 (medium-size seeders), had a high working efficiency. Therefore, the rising labor costs as a result of the low working efficiency offset the impacts of fuel-saving. Pearson’s simple correlation analysis showed that grain yield was significantly correlated with the net returns under different treatments (*r* = 0.98, *p* < 0.01 in 2017 and *r* = 0.99, *p* < 0.01 in 2018), indicating that high outputs determined high returns.

The benefit–cost ratio under zero tillage was higher than that rotary tillage in both seasons (Fig. 2e,f). Among all the seeding methods, SM1-1 resulted in a higher benefit–cost ratio in 2017, but SM3 and SM4 had higher values in 2018.

## Discussion

The present study showed that grain yield was higher under zero tillage than under rotary tillage in a soil with a high moisture content (Table 3). This type of soil increases the difficulty of tillage and leads to low tillage quality. The resulting soft and sticky soil negatively affects wheat seeding. The current investigation showed that the seeding depth was greater in the tillage soil than in the zero-tillage soil. Deep sowing increases the nutrient consumption required for seed emergence, which can result in weak seedlings. In contrast, shallow sowing decreases anaerobic stress in soils with a high water content. Furthermore, seeding near the soil surface leads to an abundance of shallow roots, which help the plants adapt to waterlogged soil^24^. In low rainfall areas, zero tillage and mulching decrease evaporation losses and maintain soil water, which promotes plant growth by alleviating drought stress^4,16^. In RWRSs, repeated soil puddling and long-term flooding for rice planting increase the soil bulk density and result in a heavy soil texture^5,6,8,25^. These soil changes adversely affect wheat growth and reduce grain yield compared to soils subjected to zero tillage and direct seeding of rice^7^. Tillage can break the hardpan and reduce subsoil compaction, with incorporated straw increasing soil porosity^10,11,13^. The tillage and increased porosity synergistically facilitate root growth and nutrient uptake^12^. Therefore, conventional tillage has been regarded as a suitable strategy after rice harvesting, regardless of the rice planting method (puddling and non-puddling) and straw management (residue incorporation and residue removal)^26^. However, it has also been reported that as zero tillage saves time (no time is spent on straw management and tillage)^14,27^, it can be used to achieve similar or even higher wheat yields than relatively late sowing after tillage^6^. In the present study, the wheat was sown on the same day, regardless of the tillage method used (tillage or zero tillage). It is worth noting that there was high soil moisture in the harvested rice fields in the present study, which differed from the experimental conditions in the previous report.

In the 2016–2017 experiments, compared to tillage, zero tillage did not increase tiller number, but improved tiller fertility, indicating more vigorous tiller growth (Table 3). A strong single stem enhances single spike yield, which contributes to achieving a high yield. In 2017–2018, zero tillage boosted the maximum tiller number, fertile tiller number, and grain yield, but did not affect tiller fertility. A comparison of the results from the two years implied that the high wet soil (95% field capacity) in 2016–2017 likely resulted in more serious damage from deficient O_2_ compared with the low wet soil (84% field capacity) in 2017–2018. It has previously been reported that water-saturated soil reduces tiller number per seedling an d inhibits root and shoot growth, affecting nutrient absorption and photosynthesis^28–31^.

Although tillage has been shown to greatly affect N absorption and remobilization^32^, the difference in N efficiency among the tillage methods can depend on soil environmental conditions. Rial-Lovera et al*.*^33^ reported that tillage influenced N use efficiency in a humid growing season. Ding et al*.*^34^ found that zero tillage achieved a higher NUpE than tillage under excessive soil moisture conditions during the early growth stage, and that there was no difference when the soil moisture was suitable for tillage. In this study, similar results were found; NUpE was higher under zero tillage than under rotary tillage when wheat was planted in harvested rice fields with high soil moisture (Table 6). It has been reported in previous studies that high NUpE is closely related to greater N accumulation, especially high N uptake after anthesis^34–38^. Some reports have indicated that N uptake during the early growth phase is not directly associated with NUpE^37,39,40^. The present study showed that the higher NUpE resulting from zero tillage was mainly attributed to greater pre-anthesis N accumulation instead of post-anthesis N accumulation (Table 6). A possible explanation for the increased pre-anthesis N accumulation is that zero tillage resulted in abundant shallow roots and enriched nutrient concentrations in the topsoil^24,41,42^. This would have facilitated the absorption of nutrients from the surface soil during the early growth period. However, zero tillage would have ultimately restricted the root growth, thus adversely affecting nutrient uptake later in the growth period^6,8,43^.

The results showed that the seeding method significantly affected grain yield, and the best method was dependent on the experimental year and tillage method. In a high wet soil (2016–2017), SM1-1 achieved the highest grain yield regardless of the tillage method (Tables 2 and 3). Compared with SM2 and SM3, the sowing depth under SM1-1 was the shallowest (near the soil surface). It has previously been reported that surface seeding could be a suitable strategy for when the soil water content is too high; surface seeding can address the potential hypoxia-related stress by encouraging topsoil root growth^14^. Besides, due to moist soil blocking the seeding port in SM1-1, the seed pipe was removed, which resulted in broadcast sowing. Compared with drill sowing, uniform (missing-row) sowing has been shown to increase the competitiveness of wheat plants via increased leaf area, tiller number, N uptake, and light interception^18,44^. These differences in the sowing method could explain why the tiller number, tiller fertility, spike number, and N uptake were greater under SM1-1 than under SM2 and SM3 (Table 3). Consequently, under the condition of a high wet soil, the small-size broadcast seeder (SM1-1) under zero tillage resulted in the sowing near the soil surface, boosting the number of early-produced tillers and their growth, achieving an improvement in grain yield through an increased spike number.

In a low wet soil (2017–2018), there were higher grain yields under SM3 and SM4 than under SM1-2 and SM2 (Table 3). This indicated that the medium-size seeders were more suitable for the 2017–2018 soil conditions than the small-size seeders. Although there were difference between the wheel compaction of SM3 and the roll compaction of SM4, this was not the critical reason leading to the differences in outputs between the two seeders. The results showed that the medium-size seeders improved tiller fertility, increased spike number with high single spike yield, and critically boosted post-anthesis N uptake (Tables 3 and 6). These results indicated that the medium-size seeders facilitated the promotion of plant development during the medium and late growth periods, possibly through better root growth resulting from deeper tillage (resulting from the medium-size seeders) and sowing. In contrast, under zero tillage, the difference in grain yield was not that marked between SM1-2, SM3, and SM4 (Table 4). This could be due to the shallow sowing of SM1-2 resulting in early-phase tiller appearance and growth, which would have increased the spike number and spike yield. However, under the condition of a low wet soil, the medium-size seeders (SM3 and SM3) under zero tillage increased fertile tillers and single-spike yield through the strong growth of tillers during the medium and late growth periods. These results indicate the complexity of selecting suitable sowing methods and the importance of considering soil moisture. Furthermore, the results demonstrate that tillage had greater effects during the early period of wheat growth, and the seeding method was more influential during the medium and late growth periods. This implies the significance of choosing synergic tillage and seeding strategies.

Previous studies have reported that zero tillage can save energy, time, and costs while increasing grain yield and achieving higher economic benefits than conventional tillage^4,14,45^. The results of the present study are consistent with these reports; the greater net return under zero tillage was attributed to the savings on tillage inputs and the increased grain yields (Tables 3 and 6 and Fig. 2). However, it has also been reported that conventional tillage increases the productivity of RWRSs compared to zero tillage^46^. Ding et al*.*^34^ considered that tillage is better than zero tillage for increasing grain yields and net returns when the soil moisture is suitable for tillage, but zero tillage facilitates the achievement of higher yield and benefits. Thus, the grain yield, as affected by the tillage method, is the determining factor underlying the economic benefit.

A similar conclusion was obtained using different seeding methods, despite the cost differences that existed between the various seeding methods. In the present study, the differences in working efficiency and fuel costs between the seeding methods were analyzed. Small-size seeders entailed similar costs to medium-size seeders due to the high labor costs associated with the low working efficiency of the small-size seeders (Figs. 2 and 3). Unfortunately, due to being restricted by the seeding area, there was only one cost investigation into the seeding methods, and it did not account for the differences caused by the tillage method. With increases in labor price and reductions in labor, medium- and even large-size seeders will become the predominant seeders used. However, the results of the present study indicated that the medium-size seeders investigated were unable to adapt to the excessive soil moisture conditions. Therefore, innovative seeding procedures and technologies must be developed urgently to address the changeable sowing conditions in harvested rice fields.

## Conclusion

In harvested rice fields with high soil moisture, zero tillage can be used to reduce the sowing depth, which facilitates early-phase wheat plant growth and N uptake, resulting in higher grain yields, NUpE, and net returns. When the wet extent of soil is high, a small-size broadcast seeder with sowing near the soil surface is recommended. In a low wet soil, a medium-size seeder is a more suitable choice for achieving higher grain yield, working efficiency, and benefits.

## Data Availability

All data generated or analysed during this study are included in this published article.
